# Variant mapping and mutation discovery in inbred mice using next-generation sequencing

**DOI:** 10.1186/s12864-015-2173-1

**Published:** 2015-11-09

**Authors:** Jabier Gallego-Llamas, Andrew E. Timms, Krista A. Geister, Anna Lindsay, David R. Beier

**Affiliations:** Department of Pediatrics, University of Washington School of Medicine, Seattle, WA USA; Center for Developmental Biology and Regenerative Medicine, Seattle Children’s Research Institute, 1900 Ninth Ave., Seattle, WA 98101 USA

**Keywords:** ENU mutagenesis, Positional cloning, NGS variant analysis

## Abstract

**Background:**

The development of powerful new methods for DNA sequencing enable the discovery of sequence variants, their utilization for the mapping of mutant loci, and the identification of causal variants in a single step. We have applied this approach for the analysis of ENU-mutagenized mice maintained on an inbred background.

**Results:**

We ascertained ENU-induced variants in four different phenotypically mutant lines. These were then used as informative markers for positional cloning of the mutated genes. We tested both whole genome (WGS) and whole exome (WES) datasets.

**Conclusion:**

Both approaches were successful as a means to localize a region of homozygosity, as well as identifying mutations of candidate genes, which could be individually assessed. As expected, the WGS strategy was more reliable, since many more ENU-induced variants were ascertained.

**Electronic supplementary material:**

The online version of this article (doi:10.1186/s12864-015-2173-1) contains supplementary material, which is available to authorized users.

## Background

Phenotype-driven screens in mice treated with chemical mutagen N-ethyl-N-nitrosurea have proven to be a powerful means to facilitate gene discovery for a wide variety of biological processes. An advantage of this method is the creation of point mutations throughout the genome in an unbiased fashion. Importantly, this has the potential to create the entire spectrum of human-disease relevant mutations, including null, hypomorphic, and gain-of-function alleles. It has been well-established that ENU treatment results in single-base changes, and the development of methods of genomic analysis has made the positional cloning of the mutant gene readily feasible.

Traditionally, the process of gene mapping in mice is done by crossing the inbred line on which the mutation was generated with a different inbred line, and following the segregation of the mutant phenotype with known informative genetic markers. This requires a two-step analysis: genetic mapping to localize the region carrying the mutation (done presently by analysis of strain-specific single nucleotide polymorphism (SNPs)), followed by sequence analysis to detect the causal variant. The development of cost-efficient whole genome (WGS) and whole exome sequencing (WES) methods has enabled genetic mapping and causal variant detection to be done in a single step. The use of WGS analysis for mutation discovery has been successful in a variety of animal models such as *C. elegans* [1], *Arabidopsis* [2, 3], zebrafish [4] and mice [4, 5]. Similarly, WES has been used with success to find causal mutations in mice [6, 7].

The application of WGS or WES for mutation analysis in ENU-induced mutant lines is particularly attractive given the evidence that most mutations caused by ENU are localized to coding regions or splice sites. While there is certainly ascertainment bias in this analysis, in the characterization of ENU mutants mapped to loci where known candidates reside, presumptively causal sequence variants can usually be found in coding regions or splice sites (e.g., [8]). Furthermore, empirical experience supports the productivity of exome analysis for characterization of ENU-induced mutations [6].

Note that in the examples cited above, abundant naturally occuring strain-specific polymorphism facilitated analysis. However, using an out-cross for the purpose of genetic mapping can in some cases be problematic, as it could introduce phenotypic variability due to the generation of a heterogeneous genetic background. Obviously, maintaining mice an on inbred background precludes the use of strain-specific informative markers. Of note, ENU treatment introduces on the order of 3000 novel heritable genetic variants [9]; even incomplete ascertainment of these in a segregation analysis should be sufficient for mutation localization, given that mapping can be reliably done with far fewer markers [10].

Bull et al. have successfully pursued such analysis for a single ENU-induced mutant, using deep WGS of multiple affected individuals [11]. In this study, we have investigated if analysis of ENU-induced sequence variants in a pooled sample of inbred mutants using WGS could reliably identify a causal mutation associated with a disease-causing phenotype. We also explored the utility of WES for this purpose, recognizing that, given exome hybridization methods generally capture ~1 % of the genome, the number of variants ascertained could preclude reliable genetic mapping.

## Results

### Generation and ascertainment of ENU mutant lines

**Fig. 1:**
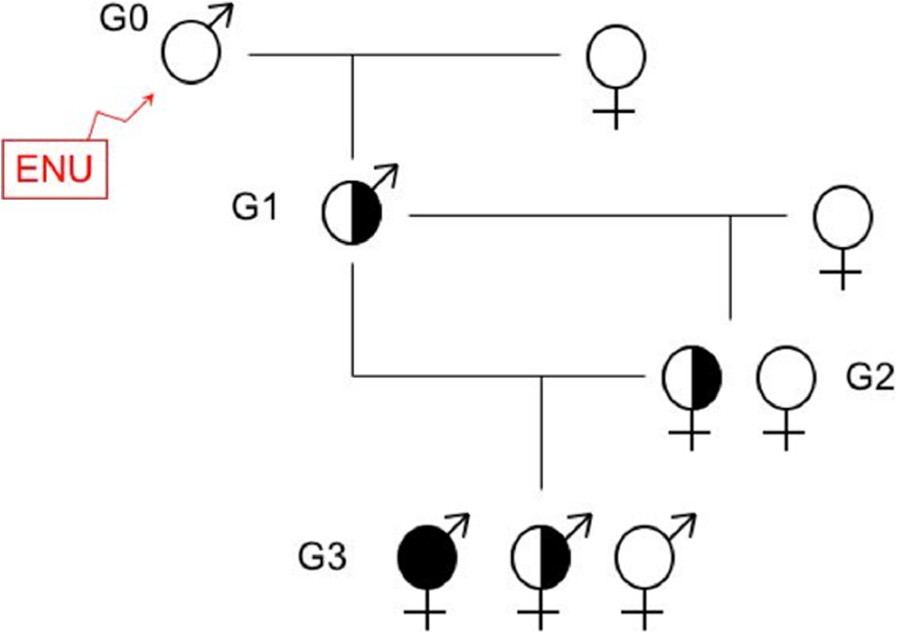
Breeding scheme for analysis of recessive mutations. G1 males have inherited a random set of de novo point mutations induced by ENU treatment of the G0 parent. The G1 founder will carry, on average, 3000 ENU-induced variants genome wide. They are crossed with wild-type females to generate G2 progeny. G2 females are backcrossed to the G1 males, and recessive mutant phenotypes are ascertained in their G3 offspring. In this breeding scheme the likelihood that a region unlinked to the mutation is homozygous is 0.125

We used our standard strategy of breeding in order to uncover mice carrying recessive mutations affecting organ development (Fig. 1) [12]. Briefly, Generation 1 (G1) males were obtained from C57BL/6 J (B6) males treated with ENU that were crossed with wild-type B6 females. These G1 males were crossed with wild-type B6 females and then crossed with their G2 daughters to generate G3 progeny. The G3 cohorts were examined for abnormal phenotypes, which are likely to be due to monogenic recessive mutations if found in multiple mice in a pedigree. In most cases the G3 mice were examined at E18.5. In some cases G3 mice were born and examined at weaning age.

For our sequencing analysis, mice were chosen from two crosses that have been maintained on an inbred background. In the first, 39 G1 males were tested by examination of a minimum of 5 G3 litters each. A total of over 3000 G3 progeny have been examined. Because only half of the G2 females will carry any mutation derived from a G1 male, a fully penetrant monogenic mutation should affect 12.5 % of the G3 progeny. 3 lines were chosen for whole genome sequencing based on phenotype: J318 (mutant frequency 8 %), J328 (mutant frequency 4.3 %), and J327 (mutant frequency 2 %). WGS analysis was also done on K416, a line with a mutant frequency of 8 % from a second inbred cross, which is ongoing. WGS analysis was done on pooled samples of at least 4 affected mice without bar-coding individual mice. For J318, J328, and J327, exome analysis was done on pools of 3 affected mice through the Broad Mutant Mouse resequencing program. A “virtual” WES analysis was done on K416 by limiting the analysis to the subset of variants identified in the WGS that were annotated as being in exons or splice sites.

### Identifying candidate phenotype-causing mutations within linked intervals

As discussed, the expectation was that we would be able to identify a large number of novel allele variants not present in the any reference genome or shared among different founders, which are presumptively ENU-induced mutations. These can then be analyzed for segregation with the mutant phenotype similar to any other informative genetic marker. The method for novel allele variant discovery is described in the Methods. This will be sensitive to sequence coverage and to filter thresholds such as read depth and quality scores. However, in all cases >2000 variants were detected for WGS (Table 1), which is ample for mapping purposes.

**Table 1:** Variant ascertainment and homozygosity mapping in four mouse mutant lines

	Mouse mutant	J318	K416	J328	J327
	Phenotype	hind limb weakness	smooth skin, barrier defect	Caudal truncation, cranio-facial defects, syndactyly	embryonic growth retardation
WGS	Samples pooled	5	4	5	4
	Genome coverage	16.6X	25.7X	14.2X	8.9X
	Number of regions of homozygosity	1	1	2	1
	Size of the homozygosity region	25 Mb	38 Mb	88 Mb/43 Mb	53 Mb
	coverage ≥ 5	90 %	89 %	83 %	80 %
	Informative SNPs	3171	3430	2032	2219
WES	Samples pooled	3	4	3	3
	Exome coverage	117X	17X^a^	121X	160X
	Number of regions of homozygosity	1^b^	1	2	1
	Size of the homozygosity region	24 Mb^b^	83 Mb	78 Mb/26 Mb	49 Mb
	coverage ≥ 5	99 %	90 %	96 %	95 %
	Informative SNPs	152	30	127	97

^a^virtual exome (see methods)

^b^Homozygous region does not contain the causal variant

Two strategies for mapping analysis were employed. Both assessed variants in sliding windows ranging from 2–20 Mb. In the first method, the percent of SNPs that were homozygous over a region was determined. In the ideal case, for a pooled sample of mice carrying a recessive mutation, a region of homozygous variants corresponding to the recombinant interval that includes the mutant locus should be ascertained. Furthermore, given the breeding strategy, the likelihood of a region unlinked to the mutation being homozygous for any one mouse is 0.125 (the likelihood that a G2 female inherits a specific allele is 0.5, so the likelihood this is homozygous in the G3 is 0.25 × 0.5); as such, the likelihood that a spurious homozygous region is identified by our analysis is 0.125^N^, where N is the number of mutants in the pool. For an N of 4 (the minimum number in our NGS analysis), this is a probability of 0.0002.

**Fig. 2:**
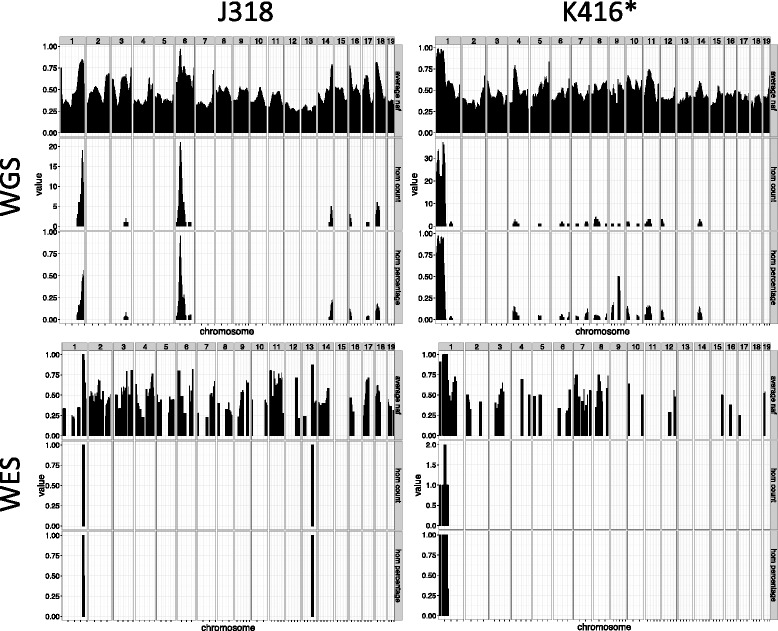
Visualization of homozygosity mapping in 2 independent mouse lines. The upper panels show homozygosity mapping using WGS data and lower panels using WES data (simulated from WGS in K416). For each line the genome was divided in 20 Mb sliding windows with a 1 Mb overlap, and three values were calculated per window; the average non-reference allele frequency for all SNPs (average naf), a count of the number of homozygote SNPs (hom count), and the percentage of SNPs that were classed as homozygote (hom percentage)

In two cases this approach worked essentially as predicted. Analysis of line J318, whose phenotype features muscle weakness, identified a region of 25 Mb in chromosome 6 (Fig. 2, Table 1); this contained 22 homozygous variants, only one of which was in a coding region or splice site (Table 2). This variant was in the gene for the chloride channel protein *Clcn1* and creates an early stop codon. The engineered null mutation of this gene has been described and has a very similar phenotype to that we observed [13, 14].

**Table 2:** Candidate causal mutations ascertained in four mouse mutant lines

Line	Average NAF threshold (20 Mb window)	gene	NAF	Chr	bp	Ref allele	Var allele	AA change	polyphen score
J318	>90 %	*Clcn1*	1	6	42291705	A	T	Stop	N/A
K416	>90 %	*Tpp2*	1	1	43971700	T	C	V549 > A	0.9
		*Stat4*	1	1	52013916	A	T	R85 > S	0.5
		*Dnah7a*	1	1	53496894	A	T	D2663 > E	0.22
		*Abca12*	1	1	71259356	A	G	Splicing	N/A
J328	>70 %	*Ssh3*	0.68	19	42265350	T	C	M316 > T	0.99
		*Macod1*	0.73	19	7066171	C	T	T160 > I	1.0
		*Pcsk5*	0.85	19	17586097	T	A	I559 > F	0.98
		*Mbl2*	0.78	19	30234044	A	G	T22 > A	0.72
		*Rbp4*	0.75	19	38118394	T	A	D193 > V	1.0
J327	>70 %	*Zbbx*	0.8	3	75085082	A	G	I254 > T	0.03
		*Adar*	0.6	3	89735676	T	C	I288 > T	0.55
		*Pex11b*	0.73	3	96643922	T	A	I241 > K	0.01

Analysis of K416 identified a 38 Mb region of homozygosity in chromosome 1 (Fig. 2, Table 1). The phenotype of this line features abnormal skin development and examination of the homozygous variants revealed exonic or splice site mutations in five genes: *Ppp1r42* (stopgain), *Tpp2* (nonsynonymous SNV), *Stat4* (nonsynonymous SNV), *Dnah7a*, (nonsynonymous SNV), *Abca12* (splice-site mutation) (Table 2). Null mutations of the latter gene have been generated and have a developmental defect in skin formation similar to that we observed [15].

In two other analyses, no regions of definitive homozygosity were identified. This could be due to a number of reasons, including the possibility that the sequenced pool included a mouse that was not homozygous, either due to a heterozygous mouse expressing the mutant phenotype, mistaken ascertainment due to a nonspecific phenotype, or by error. To address this, we used a strategy in which the novel allele frequency (NAF, which are presumptive ENU-induced mutations) were tabulated and the regions with the highest number identified. That is, variants were not scored as homozygous vs. heterozygous; rather, the relative amount of non-reference alleles in a region were scored.

**Fig. 3:**
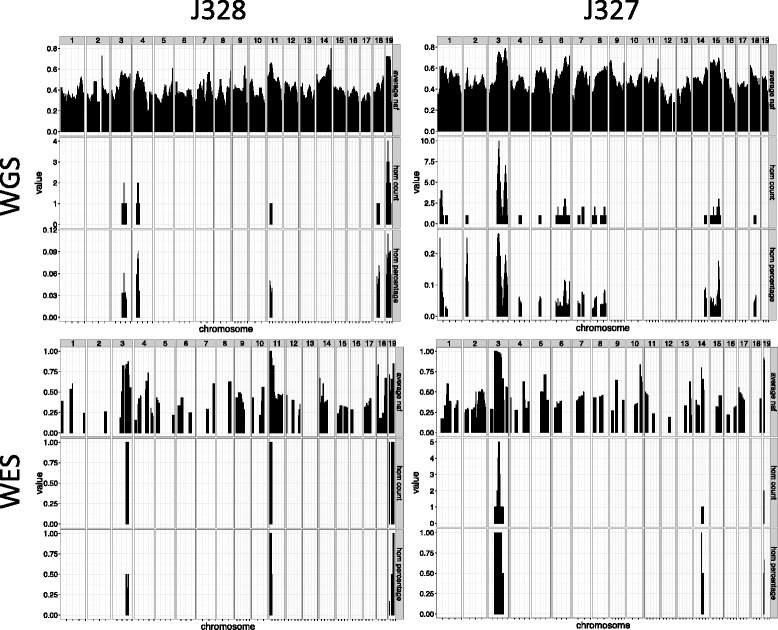
Visualization of homozygosity mapping in 2 independent mouse lines. As in Fig. 3, upper panels show homozygosity mapping using WGS data and lower panels using WES data (simulated from WGS in K416). In these cases no homozygous region was found, so regions with a novel allele frequency (naf) of >70 % were examined

For line J327, which has an embryonic growth retardation phenotype, analysis using 20 Mb windows and 70 % NAF as a cut off yielded 2 regions, one on chr 3 and one on on chr 6 (Fig. 3, Table 1). Examination of the latter revealed that these novel alleles did not skew towards homozygosity, so this region was considered unlikely to be harboring a recessive mutation. After filtering the chr 3 region for exonic, rare, good quality variants we only see 4 mutations (Table 2). These are all missense, and reside in *Zbbx*, *Adar*, *Pex11b*, and *Slc44a5*. Mice homozygous for null mutations in *Pex11b* show developmental delay and are undersized at birth [16], while mice null for *Adar* mutations are midgestation lethal with abundant apoptosis [17]. Mutants of *Slc44a5* are viable (http://www.mousephenotype.org/data/genes/MGI:3035141#section-associations) and mutants of *Zbbx* have not been reported.

In our analysis of line J328, which has a phenotype that includes caudal truncation, cardiac phenotypes, cranio-facial phenotypes, renal agenesis and an increase in the number of costal ribs, no definitive homozygous region was identified. A region of 53 Mb on chromosome 19 had a NAF >70 % (Fig. 3, Table 1); these included missense mutations in 5 genes: *Ssh3, Macrod1, Pcsk5, Mbl2* and *Rbp4* (Table 2). Null mutants of *Pcsk5* have the same spectrum of organogenesis defects as were found in line J328 [18].

As expected, because WES queries a much smaller fraction of the genome, fewer novel variants were detected and regions containing multiple homozygous variants or an elevated NAF were inconsistently identified (Table 1). In two of the four studies (J318 and J328), WES failed to identify the presumptive causal mutation ascertained using WGS. In fact, the WES analysis of J318 reported a homozygous region on a chromosome that proved to be unlinked to the causal locus. In contrast, a unique region of 5 homozygous variants included the causal mutation in the virtual WES analysis of K416, and a region of 10 homozygous variants in the WES analysis of J327 included all of the exonic mutations discovered using WGS.

### Validation of the putative causative mutation

The WGS analysis of line J318 revealed only one exonic homozygous variant in the region of homozygosity. This mutation created a nonsense mutation in the gene for the chloride channel protein, skeletal muscle (*Clcn1*). The genetically engineered null mutant for this gene has a very similar phenotype compared to the one that we observed in our animals: mutant mice exhibit mild to severe spasms of the hind limbs, abnormal hind limb reflexes, and difficulty righting [13, 14]. Sequence analysis of 11 affected mice by Sanger sequencing revealed 100 % concordance of the phenotype with the homozygous mutant genotype, and analysis of 26 phenotypically wild-type siblings identified no homozygous mice. Histological analysis of J318 mutant mice using succinate dehydrogenase staining shows the same pattern previously reported for the null mutant [19].

**Fig. 4:**
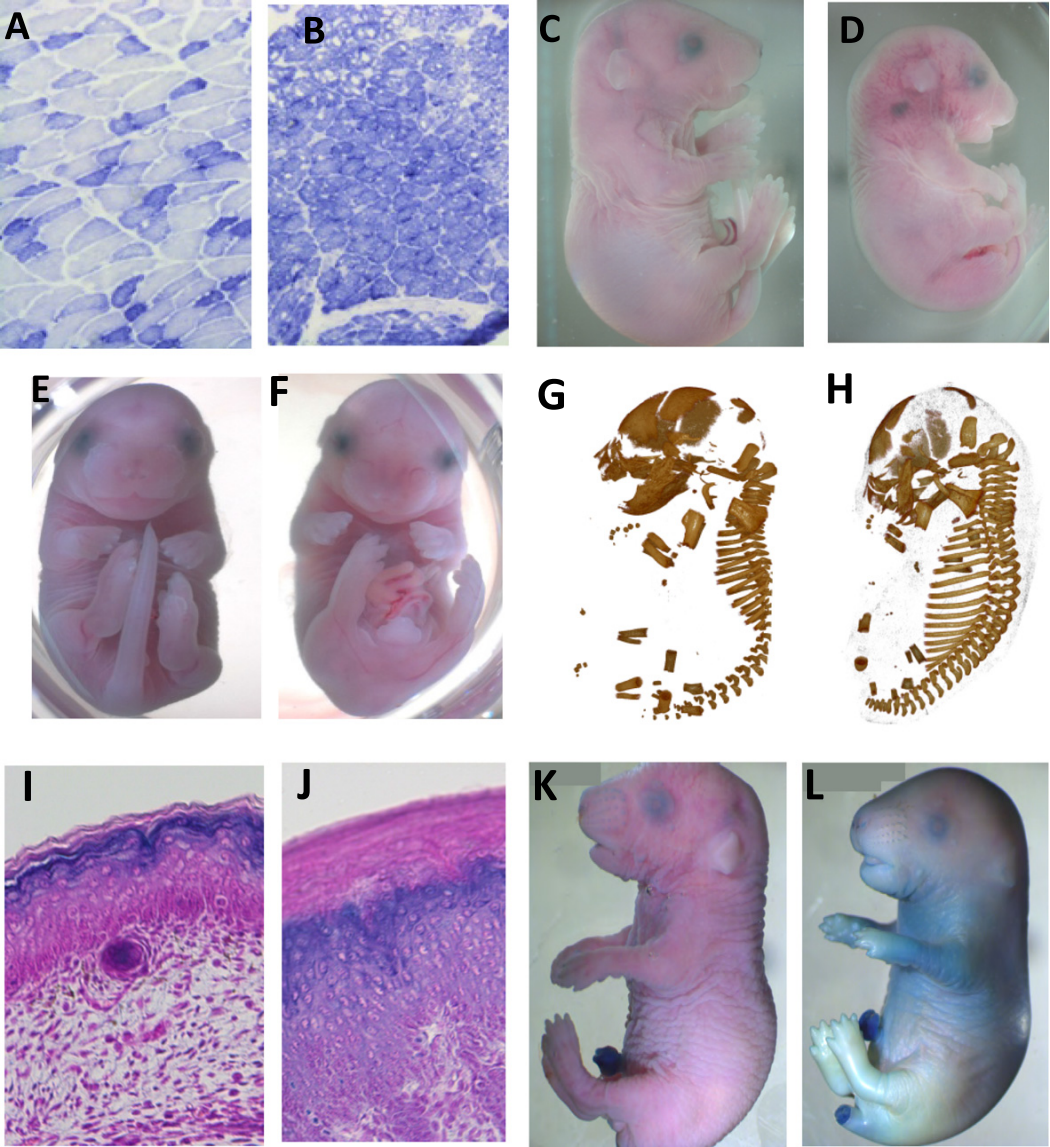
Mutant phenotype analysis. Succinate dehydrogenase staining of muscle from wild-type mice (**a**) and line J318 (**b**), which shows hind limb weakness. Compared with wild-type embryos (**c**), Line J327 mutants show growth retardation at E18.5 (**d**). Compared with wild-type e18.5 embryos (**e**), J328 mutants have a variety of abnormalities, including caudal truncation and craniofacial defects (**f**). Micro-CT images of wild-type (**g**) and a J328 mutant (**h**) shows the increase in the number of costal ribs and defects in skull development. Histological sections of body skin from a wild-type (**h**) and a Line K416 embryo (**j**) reveal hyperkeratosis in the mutant. LacZ skin permeability assay will cause skin to turn blue due to a defect in skin barrier function. Shown are representative images of a wild-type (**k**) and a Line K416 mutant (**l**)

WGS analysis of line K416 revealed 5 exonic variants in the region of homozygosity. Analysis of 14 mutants excluded *Ppp1r42*, as it was heterozygous in 4 and wild-type in 7. The mutant phenotype was completely concordant with homozygosity for the *Abca12* mutation, and of 10 phenotypically wild-type siblings, none were homozyogus. The *Abca12* mutation is in a splice-site, and analysis of the transcript reveals an abnormal transcript, which carries a premature stop mutation that truncates the protein prior to a highly conserved ABC domain (Additional file 1: Figure S1). Analysis of skin histology and skin barrier formation show abnormalities in both of these, similar to those reported for the *Abca12* null mutant mouse (Fig. 4). Given these observations, *Tpp2* and *Stat4* were not tested in the mutants by genotype analysis, as the null mutants for these genes are post-natal viable [20]. *Dnah7a* was also not tested; the null phenotype has not been reported but the Polyphen score for this mutation was 0.22.

As discussed above, WGS analysis of line J327 did not reveal a definitive region of homozygosity; rather, analysis of novel allele frequency identified a region on chr 3 containing 4 candidate exonic mutations. *Adar, Pex11b,* and *Zbbx* are tightly linked (within 21 Mb) and have not been separated by recombination in our analysis. 12 phenotypically abnormal mice were homozygous for all 3 genes, while none of their phenotypically wild-type siblings were (*N* = 35). As noted, null mutations of *Adar* and *Pex11b* are associated with developmental delay [16, 17]. However, the Polyphen score for the *Adar* missense mutation is 0.55, while that for the *Pex11b* mutation is 0.01. Null mutations of *Adar* are midgestation lethal with abundant apoptosis [17], and a TUNEL analysis of a J327 mutant liver reveals a modest but highly significant increase in apoptosis (Additional file 2: Fig. 2), perhaps indicative of this being a hypomorphic *Adar* allele. The null phenotype for *Zbbx* has not been reported; however, the Polyphen score for the *Zbbx* mutation was 0.03, suggesting it is unlikely to be causal.

For line J328, novel allele frequency analysis identified a region containing missense mutations in 5 genes: *Ssh3, Macrod1, Pcsk5, Mbl2* and *Rbp4.* Mice homozygous for null mutations in *Ssh3, Mbl2* and *Rbp4* are all post-natal viable [21–23]*.* A loss of function phenotype of *Macrod1* has not been reported. Multiple mutants were tested and only the variant present in the *Pcsk5* gene was present in all of the affected mice. Also, the *Pcsk5* variant had a Polyphen score of 0.979, and the phenotype of this line is identical to that described for the null allele [18], including such specific features as the increased number of costal ribs (Fig. 4).

## Discussion

We and others have described methods in which whole genome sequencing can be used for positional cloning of mutations. In most cases these rely on linkage analysis using naturally-occuring genetic variants. These will be present in a mutant because the mutation was present on a non-inbred genetic background, or because informative genetic variation was purposefully introduced by breeding to a different inbred strain. In both cases, the mutant samples will have abundant genetic heterogeneity, which may result in phenotypic variation.

We hypothesized that for a mutant line obtained from a mutagenesis experiment, one might use the induced variants for the purpose of genetic mapping. This would enable analysis on a relatively homogeneous genetic background, as well as avoid the need for an outcross for genetic mapping. The requirements for this would be that the induced variants be abundant, randomly distributed across the genome, and readily scored. Treatment of mice with a fractionated dose regimen of ENU has now been well documented to create single base mutations at a frequency of approximately 1/Mb, and bioinformatic tools for their identification and allele frequency measurement are robust.

To test this, we performed sequencing analysis on 4 different mutant lines obtained from a screen of mutagenized C57BL/6 J mice that were maintained on this background. Whole genome sequence analysis was done on pooled samples. We also obtained whole exome data on pooled samples for 3 of these lines, and created a virtual exome sample from the fourth.

In two cases the strategy worked exactly as predicted—thousands of novel sequence variants were discovered, of which a small number were homozygous and closely linked. Despite the fact that the mutant pool was small (*N* = 4 or 5) this region was of relatively modest size, which has been our experience in mapping studies using outcrossed samples [10]. In both cases exonic mutations were readily found in the presumptive recombinant interval, and were found to be concordant with the abnormal phenotype in other mutant mice from the same line. In both cases null mutations of the candidate mutant gene had been characterized and had the same phenotype as that obtained by us.

In two cases the strategy was modified because no region of definitive homozygosity was found. This could be true for a number of reasons. For example, some mutations will result in a phenotype even when heterozygous (e.g. Szumska et al. described that in rare cases the heterozygote disruption of the gene *Pcsk5* has a phenotype [18]). Alternatively, some phenotypes will occur independently of the mutation being characterized due to background effects (e.g. the A/J strain has a high incidence of cleft lip/palate). Errors in sample handling or identification may also occur. To accommodate these possibilities we identified regions which had a higher frequency of non-reference variants compared to the random (50 %) distribution expected in unlinked regions. This served to identify candidate regions which were then further tested in independent individual affected mice. This strategy worked well, and candidate genes for the mutant loci were found in both cases. A caveat is that the single base changes induced by ENU are well-known to cause both hypomorphic and neomorphic alleles. The possibility that the phenotype of an ENU-induced mutant will be different than that reported for a null mutant allele should be considered in the examination of candidate variants.

We also compared the results obtained using WGS to that obtained by analysis of WES data. As expected, the latter approach can serve to identify causal mutations in pooled samples. However, the much smaller number of variants ascertained reduces the mapping effectiveness of the approach; that is, when a region with a large number of homozygous variants or a high novel allele frequency is found using the WGS approach, one has considerable confidence that the mutation is resident in that region. Further, while it is the case that ENU-induced variants are usually found in sequences queried by exome analysis, this may not always be true, and having the entire cohort of novel variants in a recombinant interval may be useful. This of course needs to be balanced against the higher cost of WGS analysis compared to WES analysis.

The characterization of ENU-induced mutation on an inbred background was first reported by Bull et al. [11]. Our method has a number of advantages; most importantly, by using a pooling approach we have proven that less sequencing can be used to confidently identify both a region of homozygosity and a causal variant (although Bull and colleagues did indeed predict by modeling that less dense coverage could be used). Additionally, our method does not require all affected mice to be from the same G2 pair, and it has been shown to identify disease mutations even when all sequenced mice do not share a homozygous region. Wang et al. have recently shown the feasibility of using WES of founder strains as a mean to discover new ENU-induced mutations [24]. This approach has the virtue of discovering all of the potential causal mutations in a screen in single step. However, it does require the generation of SNP assays for a large number of the induced variants in order to perform the linkage analysis of the mutant mice. What is now evident is that ENU-induced variants can be effectively used as informative markers for genetic analysis on inbred backgrounds; the specific strategy chosen will depend on the scope of the screen and the resources available.

Of note is that in several cases our analysis identified multiple mutant loci, which then had to be individually assessed as candidate loci. This ambiguity is due to the constrained genetic resolution of our analysis, which is a function of read depth (in that heterozygous markers that define recombinant breakpoints are incompletely ascertained) and pool size (in that a modest number of meioses were queried). For WGS analysis, high resolution analysis could be obtained by increasing the latter, although this may require a concomitant increase in the former to reliably identify heterozgyous markers. For WES analysis, resolution is likely to be limited by the modest numbers of informative variants that can be obtained by exome capture.

## Conclusions

What is clear is that NGS on pooled samples (or multiple individual bar-coded samples) can be used for the simultaneous identification of ENU-induced variants, ascertainment of regions of non-random allele distribution, and causal mutation discovery. This enables positional cloning studies that do not depend on foreknowledge of informative markers, or, for analysis on inbred mice, even their existence. This latter capability is important, as it allows for mutagenesis screens on homogenous genetic backgrounds, removing strain-specific phenotypic variation as a confounder. Application of this approach is particularly useful for characterization of quantitative traits, or for analysis of modifying loci that affect the phenotype of a sensitizing mutation. One can contemplate using this approach even for complex phenotypes such as behavior. However, the key to any screening analysis is a rigorous definition of phenotype, such that the presumptive mutants chosen for NGS analysis are highly likely to be segregating the causal mutations.

## Methods

### Mutant mouse generation

Mutant mouse strains were generated by treating C57BL/6 J mice with the mutagen ENU as previously described (reviewed in [25]). Adult male animals received 90 mg of ENU per kilogram of body weight over three weekly intraperitoneal injections. Once fertility was recovered, the animals were mated with untreated C57BL/6 J females to generate G1 offspring. The G1 males were crossed with C57BL/6 J females and their G2 daughters were backcrossed to the G1 male to generate G3 mice. These were usually examined just before birth (e18.5); some G3 cohorts were allowed to be born and were examined prior to weaning.

### DNA extraction

DNA for high-throughput sequencing was isolated from spleen using a Qiagen DNeasy Blood and Tissue Kit (Qiagen, Santa Clarita, CA USA) or by phenol/chloroform extraction. Pools of 3 to 5 samples worth of extracted DNA were then send for library preparation in order to complete a whole genome or a whole exome sequencing (WGS, WES) at the HudsonAlpha institute for biotechnology, Huntsville, Alabama 35806.

### Sequencing data analysis: Mapping, SNP calling and annotation

Paired end sequencing data were mapped to the last reference genome mm10 using BWA-MEM (Burrows-Wheeler alignment tool) [26] using default parameters. The Genome Analysis Toolkit (GATK) [27] was used to realign reads around known indels, and recalibrate quality scores to reduce artifacts caused by the sequencing process/chemistry. Picard (Broad Institute: http://broadinstitute.github.io/picard/) was used to mark the duplicate reads. SNPs were then identified using the SAMtools suite (mpileup, bcftools) [28]. All SNPs were annotated with ANNOVAR [29] using refGene as a gene model.

To identify the ENU induced SNPs for homozygosity mapping with the highest confidence, we applied additional filtering. SNPs were removed if; they were present in publically available databases (dbSNP, Sanger Mouse exomes) or in-house controls, located within a repeat region (repeat masker), had low likelihood of being real (<Q30), had low read depth (<5x), or if they were multiallelic. Homozygosity mapping was carried out using generated Python scripts and the Python version of BEDTools [30] (Pybedtools) [31]. The genome was divided into sliding windows of 2, 5, 10 or 20 Mb with a 1 Mb overlap. We then calculated a number of matrices per window using the filtered SNPs that should identify homozygous regions; count of SNPs defined as homozygous, the percentage of SNPs defined as homozygous, the average non-reference allele frequency. The results were plotted with the data on the X and Y chromosome being removed as the breeding strategy excludes the maintenance of X-linked or Y-linked mutations.

To identify potential causal ENU mutations, SNPs were filtered as previously described, with additional filtering being applied to identify SNPs in splice sites (within 10 bps of an exon) or non-synonymous SNPs in coding regions, and by zygosity.

### Validation

Candidate mutations were validated by PCR amplification and sequencing of affected and unaffected litter mates. Sequencing data were analyzed using Sequencer 5.0 (gene Codes Corp, Ann Arbor, Mi, USA). Primers for PCR amplification were designed using Primer3 software.

### Histology

Muscle tissue was frozen using isopentane that was refrigerated in liquid nitrogen and stored at −80 °C. Samples were sectioned in a cryostat producing 5 μm sections that were stained with haematoxylin and eosin or 20 μm sections tested for mitochondrial respiratory function using Succinate Dehydrogenase [32]. Briefly, sections were incubated in 1.5 mM NBT, 130 mM sodium succinate, 0.2 mM PMS and 1.0 mM sodium azide in 0.1 M PBS pH7.0 for 40 min at 37 °C. Sections were then washed in PBS and dehydrated in increasing concentration of ethanol and then incubated in Xylene.

Embryos used for skin analyses were fixed in Bouin’s fixative at least 24 h, embedded in paraffin, sectioned, and stained with hematoxylin and eosin.

### Skin permeability assay

Embryos were collected at E18.5 and stained with X-gal (Life Technologies) according to a standard protocol (Indra and Leid, 2011, PMID: 21874444).

### Micro-CT

Samples were imaged at lower resolutions with a SkyScan 1174 scanner (http://www.skyscan.be) employing a 50 keV/40 W tungsten x-ray source and a 1.3 megapixel CCD camera. This scanner also has variable optical magnification of the scintillator panel, giving fields of view from 6 mm to about 30 mm, with voxel sizes from 6 μm to about 30 μm and actual spatial resolution limits from about 15 to 75 μm. All images were reconstructed using the software provided with the microCT systems. Images were visualized using the Drishti software (publically available free software). The SkyScan 1174 images were reconstructed without binning and were stored as BMP image stacks.

### Detection of apoptosis

Frozen whole embryo sections were subjected to TUNEL staining using an *In Situ* Cell Death Detection Kit (Roche Applied Science). Direct fluorescein labeling during the TUNEL reaction was used for detection of apoptotic cells. Hoechst labelling was used as a counterstain. Livers from each section were use to count the apoptotic cells.

#### Ethics

Mouse studies were reviewed and approved by the Seattle Children’s Research Institute Institutional Animal Care and Use Committee.

## Additional files

Additional file 1: Figure S1. Sequence analysis of transcripts from K416 mutant mice reveals a mixed population that includes wild-type and mutant versions carrying a 4 base pair insertion that changes the frame and results in premature termination. (PDF 38 kb) Additional file 2: Figure S2. TUNEL analysis of 8 fields each from liver samples from E18.5 wild-type and J327 mice reveal a modest but highly significant increase in apoptosis in the mutant (*p* = 7.06E-05). (PDF 21 kb)
