# Supplementary material for: Variant mapping and mutation discovery in inbred mice using next-generation sequencing
Source: BMC Genomics. 2015 Nov 9;16:913. doi: 10.1186/s12864-015-2173-1 (PMC4640199; doi:10.1186/s12864-015-2173-1)
Supplement: Additional file 1: Figure S1. — Sequence analysis of transcripts from K416 mutant mice reveals a mixed population that includes wild-type and mutant versions carrying a 4 base pair insertion that changes the frame and results in premature termination. (PDF 38 kb) [file 12864_2015_2173_MOESM1_ESM.pdf]

Supplemental Fig. 1

```
Abca12 Mutant CATGGGATTCCAGAGAAGGACATCAAAGACGCAAACTGTTCATAAACTCCTTAGGA
              ||||||||||||||||||||||||||||||    ||||| |||||||||||||||||
Reference     CATGGGATTCCAGAGAAGGACATCAAAGAC----ACTGTCCATAAACTCCTTAGGA
                  Abca12 EXON 47                     Abca12 EXON 48
```
