# Supplementary figures and images for: Variant mapping and mutation discovery in inbred mice using next-generation sequencing
Source: BMC Genomics. 2015 Nov 9;16:913. doi: 10.1186/s12864-015-2173-1 (PMC4640199; doi:10.1186/s12864-015-2173-1)

Supplemental Fig. 2

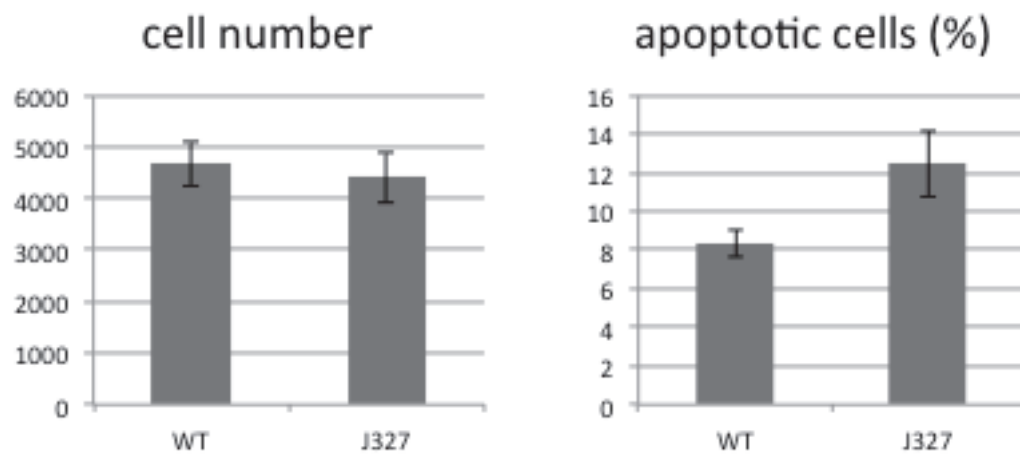

Supplement: Additional file 2: Figure S2. — TUNEL analysis of 8 fields each from liver samples from E18.5 wild-type and J327 mice reveal a modest but highly significant increase in apoptosis in the mutant (p = 7.06E-05). (PDF 21 kb) [file 12864_2015_2173_MOESM2_ESM.pdf]
